# Metabolomic signature of mouse cerebral cortex following *Toxoplasma gondii* infection

**DOI:** 10.1186/s13071-019-3623-4

**Published:** 2019-07-29

**Authors:** Jun Ma, Jun-Jun He, Jun-Ling Hou, Chun-Xue Zhou, Fu-Kai Zhang, Hany M. Elsheikha, Xing-Quan Zhu

**Affiliations:** 1 State Key Laboratory of Veterinary Etiological Biology, Key Laboratory of Veterinary Parasitology of Gansu Province, Lanzhou Veterinary Research Institute, Chinese Academy of Agricultural Sciences, Lanzhou, 730046 Gansu People’s Republic of China; 2 Department of Parasitology, Shandong University School of Basic Medicine, Jinan, 250012 Shandong People’s Republic of China; 3 Faculty of Medicine and Health Sciences, School of Veterinary Medicine and Science, University of Nottingham, Sutton Bonington Campus, Loughborough, LE12 5RD UK

**Keywords:** Metabolomics, Neuropathy, Cerebral cortex, *Toxoplasma gondii*, Host-parasite interaction, Metabolism

## Abstract

**Background:**

The protozoan parasite *Toxoplasma gondii* infects and alters the neurotransmission in cerebral cortex and other brain regions, leading to neurobehavioral and neuropathologic changes in humans and animals. However, the molecules that contribute to these changes remain largely unknown.

**Methods:**

We have investigated the impact of *T. gondii* infection on the overall metabolism of mouse cerebral cortex. Mass-spectrometry-based metabolomics and multivariate statistical analysis were employed to discover metabolomic signatures that discriminate between cerebral cortex of *T. gondii*-infected and uninfected control mice.

**Results:**

Our results identified 73, 67 and 276 differentially abundant metabolites, which were involved in 25, 37 and 64 pathways at 7, 14 and 21 days post-infection (dpi), respectively. Metabolites in the unsaturated fatty acid biosynthesis pathway were upregulated as the infection progressed, indicating that *T. gondii* induces the biosynthesis of unsaturated fatty acids to promote its own growth and survival. Some of the downregulated metabolites were related to pathways, such as steroid hormone biosynthesis and arachidonic acid metabolism. Nine metabolites were identified as *T. gondii* responsive metabolites, namely galactosylsphingosine, arachidonic acid, LysoSM(d18:1), l-palmitoylcarnitine, calcitetrol, 27-Deoxy-5b-cyprinol, l-homophenylalanine, oleic acid and ceramide (d18:1/16:0).

**Conclusions:**

Our data provide novel insight into the dysregulation of the metabolism of the mouse cerebral cortex during *T. gondii* infection and have important implications for studies of *T. gondii* pathogenesis.

**Electronic supplementary material:**

The online version of this article (10.1186/s13071-019-3623-4) contains supplementary material, which is available to authorized users.

## Background

*Toxoplasma gondii* is a worldwide prevalent pathogen with global seropositivity rates that range from 0 to over 90% [1]. *Toxoplasma gondii* has an indirect, multistage life-cycle that involves tachyzoites, bradyzoites-containing cysts and oocysts. This parasite has an exceptionally wide range of intermediate hosts including almost all warm-blooded vertebrate animals and humans. Tachyzoites and bradyzoites occur in the tissues of the intermediate hosts. However, members of the family Felidae are the only animals that can serve as the definitive host of *T. gondii*, where gametogony occurs in their intestine, resulting in the formation and excretion of oocysts in the feces. Ingestion of infectious oocysts with contaminated food or water, or eating improperly cooked or raw meat containing tissue cysts are the two main routes of *T. gondii* infection in humans.

*Toxoplasma gondii* establishes a quiescent infection with little to no clinical disease in most immunocompetent individuals. However, this parasite is responsible for a range of devastating sequelae in immunocompromised patients and in developing fetuses if their mothers acquire infection during pregnancy [2, 3]. In recent years, the impact of *T. gondii* infection on brain neurochemistry and the subsequent neuropsychiatric consequences has received significant attention [4, 5]. Additionally, behavioral abnormalities have been observed in murine hosts in association with *T. gondii* infection [6, 7]. Disruption of glutamate metabolism, and glutamatergic and GABAergic signaling pathways in the brain are some of the mechanisms used by *T. gondii* to alter the behavior of infected hosts [5, 8]. *Toxoplasma gondii* parasites can also alter the regulation of other neuroactive substances, such as dopamine [9], quinolinic acid [10] and kynurenine [11].

Previous transcriptomics [12–14] and proteomics [15–17] studies have revealed some mechanisms by which *T. gondii* can alter gene expression and protein abundance in the brain of the affected hosts. Additionally, liquid chromatography–mass spectrometry (LC–MS)-based metabolomics analysis revealed altered metabolism of key molecules and regulatory pathways in the brain, spleen, liver, and serum of mice following *T. gondii* infection [18–21]. Given the complexity of brain structure and the wide spectrum of the neuropathological changes associated with *T. gondii* infection, it is of great interest to determine specific metabolic signature associated with *T. gondii*, particularly in key brain regions such as the cerebral cortex where *T. gondii* cysts are often found [22, 23].

Here, a LC–MS-based metabolomics approach was used to analyze the global metabolic alterations induced in the cerebral cortex of mice following *T. gondii* infection. Our findings reinforce previous findings [21] and provide new insights into *T. gondii* neuro-pathogenesis.

## Methods

### Animal infection and cerebral cortex collection

Mice were infected with *T. gondii* PRU strain (genotype II) to produce tissue cysts that were subsequently used for the infection experiments. A total of 36 female BALB/C mice, 3-weeks old, were purchased from Lanzhou University Laboratory Animal Center (Lanzhou, China). Mice were allocated into six groups with 6 mice per group: 7T (infected group at 7 dpi); 7C (control group at 7 dpi); 14T (infected group at 14 dpi); 14C (control group at 14 dpi); 21T (infected group at 21 dpi); and 21C (control group at 21 dpi). The mice in infected groups were challenged by oral gavage with 10 cysts of *T. gondii* PRU strain suspended in 0.5 ml PBS, whereas mice in the control groups were mock-treated with an equal volume of PBS only. All mice were provided non-medicated feed and water ad libitum throughout the experiment. At 7, 14 and 21 dpi, mice from each sampling time were humanely sacrificed by CO_2_ asphyxiation. Cerebral cortex of each mouse was dissected with scissors and forceps, and washed immediately with PBS to remove the blood. The isolated cerebral cortices were stored frozen at − 80 °C until use.

### Detection of *T. gondii* infection in the cerebral cortex of mice

Equal parts of the collected cerebral cortices were used for DNA extraction. DNA of each cerebral cortex sample was extracted using a TIANamp Genomic DNA kit (TianGen, Beijing, China) according to the manufacturer’s instructions. *Toxoplasma gondii* infection was detected by PCR using primers that target the *B1* gene (F: 5′-TGC ATA GGT TGC AGT CAC TG-3′ and R: 5′-TCT TTA AAG CGT TCG TGG TC-3′). The amplification conditions were: 95 °C for 5 min followed by 35 cycles of 95 °C for 10 s, 60 °C for 10 s and 72 °C for 20 s. A negative control template (PBS) was included in each PCR run to exclude the false positive results. Positive amplified fragments were submitted to the Genewiz company (Beijing, China) for sequencing in both directions.

### Metabolite extraction

The frozen cerebral cortices were taken out of the − 80 °C freezer and kept at − 20 °C for 30 min, and then at 4 °C to defrost gradually. Approximately 25 mg of each defrosted tissue sample were used for metabolite extraction by mixing with 800 μl of H_2_O/MeOH (50:50% v/v) and lysis using TissueLyse bead-mill homogenizer (Qiagen, Hilden, Germany). The cerebral cortex homogenates were centrifuged at 25,000×*g* for 20 min at 4 °C. Supernatants were transferred into new tubes and 50 μl of each supernatant were filtered using a SPE column (Strata TM-X 33 μm polymeric reversed phase column, Phenomenex, Torrance, CA, USA) for solid phase extraction, and the soluble metabolites were dissolved in acetonitrile. Approximately 20 μl from each sample were pooled together as a QC sample and used to assess the reproducibility and reliability of the LC–MS method. The extracted metabolites were stored at − 80 °C until use.

### LC–MS/MS analysis

The soluble metabolomics (including hydrophobic molecules) of all samples were analyzed. All chromatographic separations were performed using an ultra-performance liquid chromatography (UPLC) system (Waters, Manchester, UK). An ACQUITY UPLC BEH C18 column (100 × 2.1 mm, 1.7 μm; Waters) was used for the reversed phase separation. The column oven was maintained at 50 °C. The flow rate was set at 0.4 ml/min and the mobile phase consisted of solvent A (water + 0.1% formic acid) and solvent B (acetonitrile + 0.1% formic acid). A gradient elution process was performed to elute metabolites as follows: 100% solvent A (water + 0.1% formic acid) for 0–2 min; 0–100% solvent B (acetonitrile + 0.1% formic acid) for ~ 11 min; 100% solvent B for 11–13 min; 100% solvent A for 13–15 min. The eluted metabolites were analyzed using a high-resolution tandem mass spectrometer, SYNAPT G2 XS QTOF (Waters). For both positive and negative ion modes, the capillary and sampling cone voltages were set at 2 kV and 40 V, respectively. The TOF mass range ranged from 50 to 1200 Da and the scan time was 0.2 s. For the MS/MS detection, all precursors were fragmented using 20–40 eV, and the scan time was 0.2 s. During the acquisition, the LE signal was acquired every 3 s to calibrate the mass accuracy. Centroid MSE (mean square error) mode was used for the collection of mass spectrometry data.

### Metabolite identification and multivariate statistical analysis

The raw data were imported into the software Progenesis QI v. 2.2 for peak detection and alignment. The assigned modified metabolites were identified by searching the HMDB database (http://www.hmdb.ca/spectra/ms/search) using Progenesis QI. The molecular mass data (m/z) and retention time (min) were used to identify the metabolites. The putative metabolites were mapped to the Kyoto Encyclopedia of Genes and Genomes (KEGG; http://www.genome.jp/kegg/) by matching the *m*/*z* of our samples with those from the database. The pre-processed data were imported to *metaX*, a R package software for statistical analysis. Student’s *t*-test was used to identify significantly different metabolites between infected and control mouse groups, and *P* < 0.05 was considered significant. *P*-values were adjusted for multiple testing using the Benjamini–Hochberg method at a false discovery rate threshold of 5%. The dataset was also subjected to unsupervised multivariate statistical analyses, including principal component analysis (PCA) and partial least-square discriminant analysis (PLS-DA), using SIMCA v.13.0 software. *Toxoplasma gondii* responsive metabolites were identified using receiver-operating characteristic (ROC) curve analysis by calculating the area under the curve (AUC) using the *pROC* package in R. Cytoscape v.3.3 was used for visualization of relationship among metabolites and pathways.

## Results

### Confirmation of infection

PCR coupled with sequencing analysis confirmed that all cerebral cortices collected from infected mice at 7, 14 and 21 dpi were *T. gondii* B1 gene positive (Additional file 1: Figure S1). No positive amplification products of *T. gondii* B1 gene were detected in cerebral cortices of mice in the control groups (Additional file 1: Figure S1). At 14 dpi, mice in infected groups showed clinical signs such as anorexia, hunched back and ruffled fur. None of these clinical signs were observed in the control mice at 14 dpi. At 21 dpi, mice in infected group seemed to regain their normal physical status.

### Infection-related metabolic changes

**Fig. 1:**
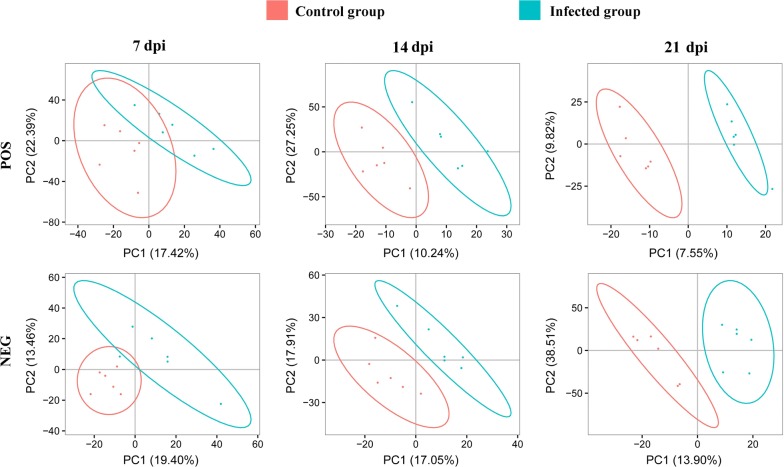
Unsupervised multivariate analysis of the global metabolic changes in the cerebral cortices of mice. Partial least squares projection to latent structures discriminant analysis (PLS-DA) score plots of the first two principal components (PC1 and PC2) showing the effect of infection on the metabolic profile of cerebral cortices at 7, 14 and 21 days post-infection (dpi). Distinct metabolic differences were observed between infected and control mouse groups particularly at 14 and 21 dpi. POS and NEG indicate positive ion mode and negative ion mode, respectively

A total of 3200 ions were detected in the negative electrospray ionization (ESI−) mode and 1436 metabolites were putatively identified, whereas 6198 ions were detected in the positive electrospray ionization (ESI+) mode with 3952 metabolites putatively identified. PLS-DA analysis was performed to identify the differences in the metabolic profile between infected and non-infected cerebral cortices. As shown in Fig. 1, in both positive and negative ion modes, the metabolites of infected and control cerebral cortices were clustered in separate groups. Additionally, as the infection developed at 14 and 21 dpi, the differences and separation between infected and non-infected cerebral cortices increased. This result indicates that the metabolic patterns of the infected cerebral cortices are distinct from those of non-infected cerebral cortices.

### Time-dependent variation in the differentially abundant metabolites

**Fig. 2:**
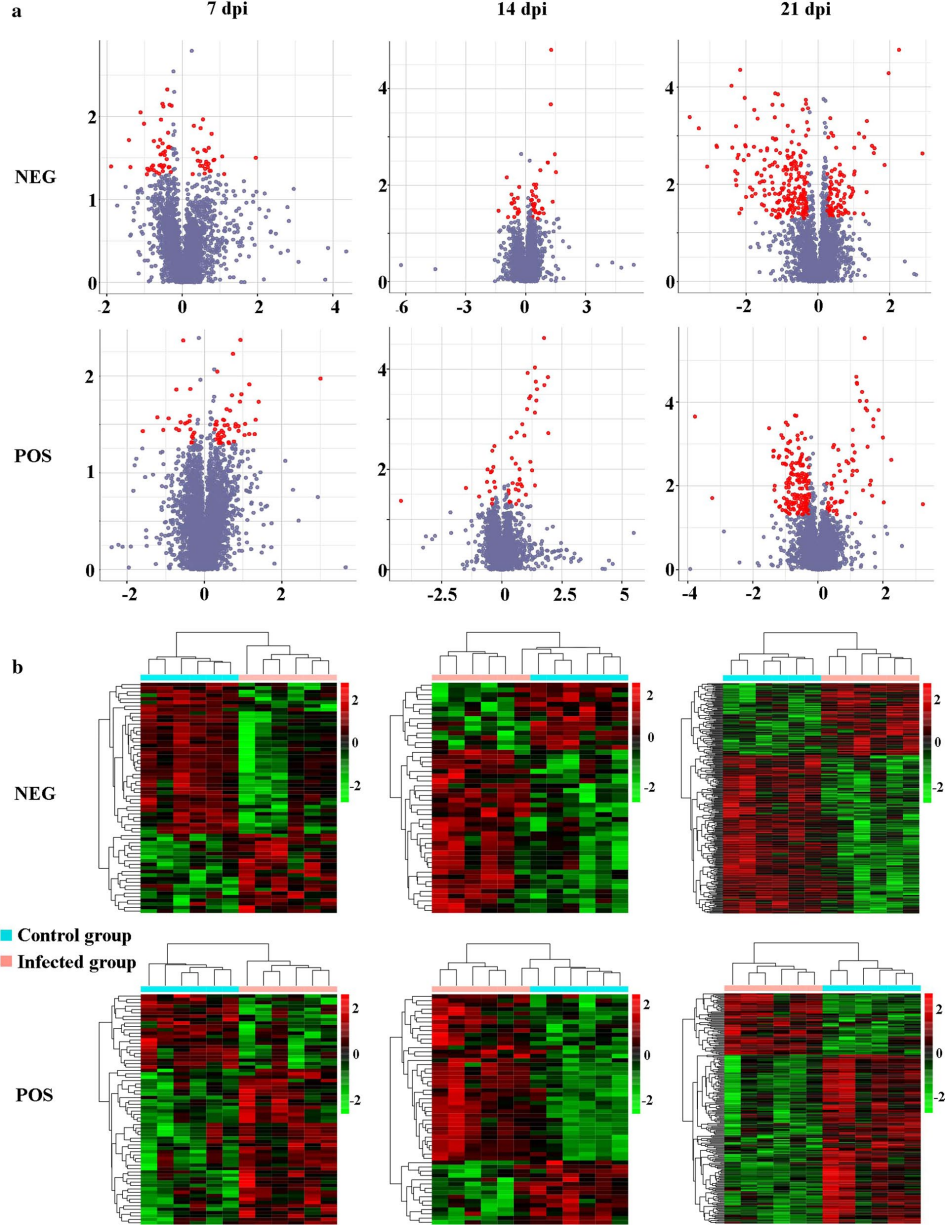
Metabolic differences in infected cerebral cortices at 7, 14 and 21 dpi. **a** Volcano plots of the differentially abundant metabolites. The log_2_ fold change is shown on the x-axis. Statistical significance displayed by − log10 (*P*-value) is shown from 0 to 4 on the y-axis. Metabolites having a fold change of > 1.5 and *P* < 0.05 (Student’s *t*-test) are represented by red dots. Metabolites that were not significantly changed are denoted by blue dots. Fold changes of the infected samples relative to the untreated control are based on the mean values of six biological replicates per group. **b** Heatmaps showing significantly dysregulated metabolites in the mouse cerebral cortex. Upregulated (red) or downregulated (green) metabolites (fold change > 1.5; *P *< 0.5) are indicated. POS and NEG indicate positive ion mode and negative ion mode, respectively

**Fig. 3:**
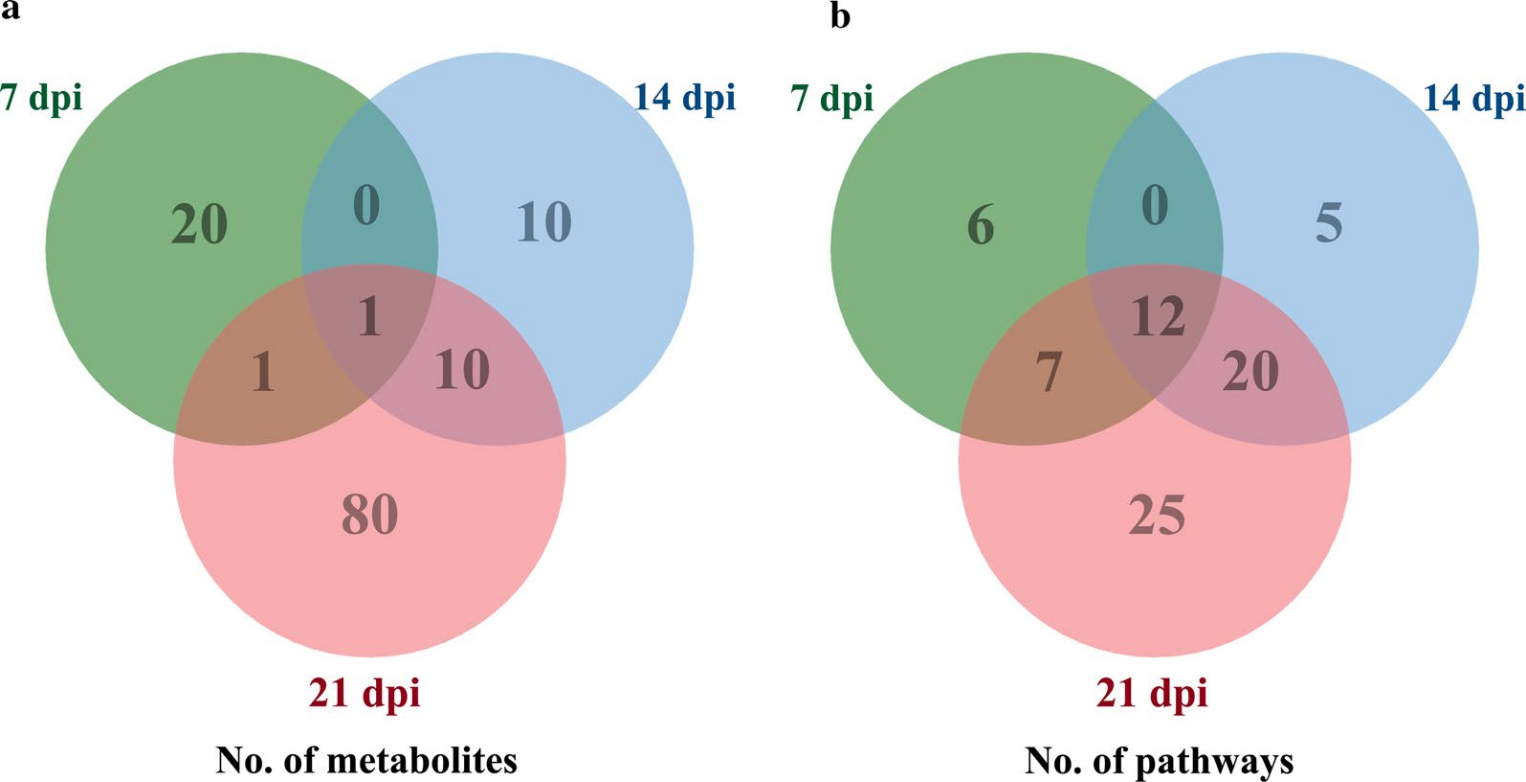
Venn diagrams showing the number of **a** common and unique differentially abundant metabolites between mouse groups and **b** metabolic pathways significantly affected following infection with *T. gondii* at 7, 14 and 21 dpi

To identify the temporal differences between infected and non-infected cerebral cortices, the differences in the metabolites between *T. gondii*-infected groups and non-infected groups were analyzed at 7, 14 and 21 dpi. Dozens of differentially abundant metabolites exhibited altered levels in *T. gondii*-infected cerebral cortices. In the negative ionization mode, 64, 48 and 288 metabolites showed differential abundances at 7, 14 and 21 dpi, respectively. Furthermore, 68, 54 and 229 metabolites were differentially abundant in the positive ionization mode. The volcano and heat map plots of these metabolites are shown in Fig. 2. The details of the differentially abundant metabolites that were mapped to KEGG pathways are shown in Additional file 2: Table S1. Our analysis revealed that 22, 21 and 92 differentially abundant metabolites were mapped to KEGG pathways at 7, 14 and 21 dpi, respectively. Additionally, only 20, 10 and 80 differentially abundant metabolites were identified at 7, 14 and 21 dpi, respectively (Fig. 3a). 1-Acyl-sn-glycero-3-phosphocholine was the only differentially abundant metabolite that was altered at all three time points, where it was upregulated at 7 and 14 dpi, and downregulated at 21 dpi (Additional file 2: Table S1).

### Metabolic pathways affected by *T. gondii*

**Fig. 4:**
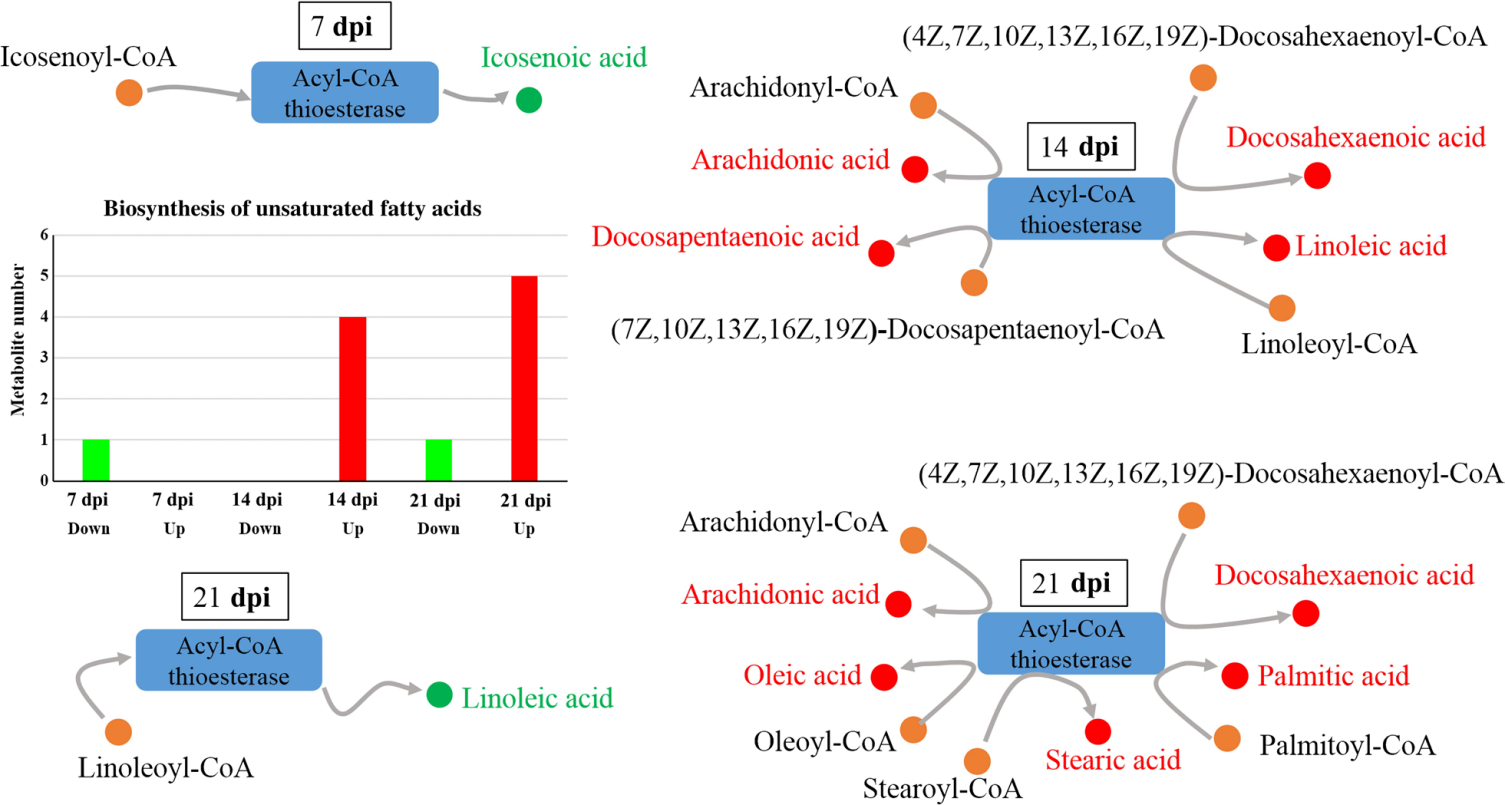
Differential regulation of the metabolites involved in the biosynthesis of unsaturated fatty acids pathway at 7, 14 and 21 dpi. Red and green colors represent upregulated and downregulated metabolites, respectively

**Fig. 5:**
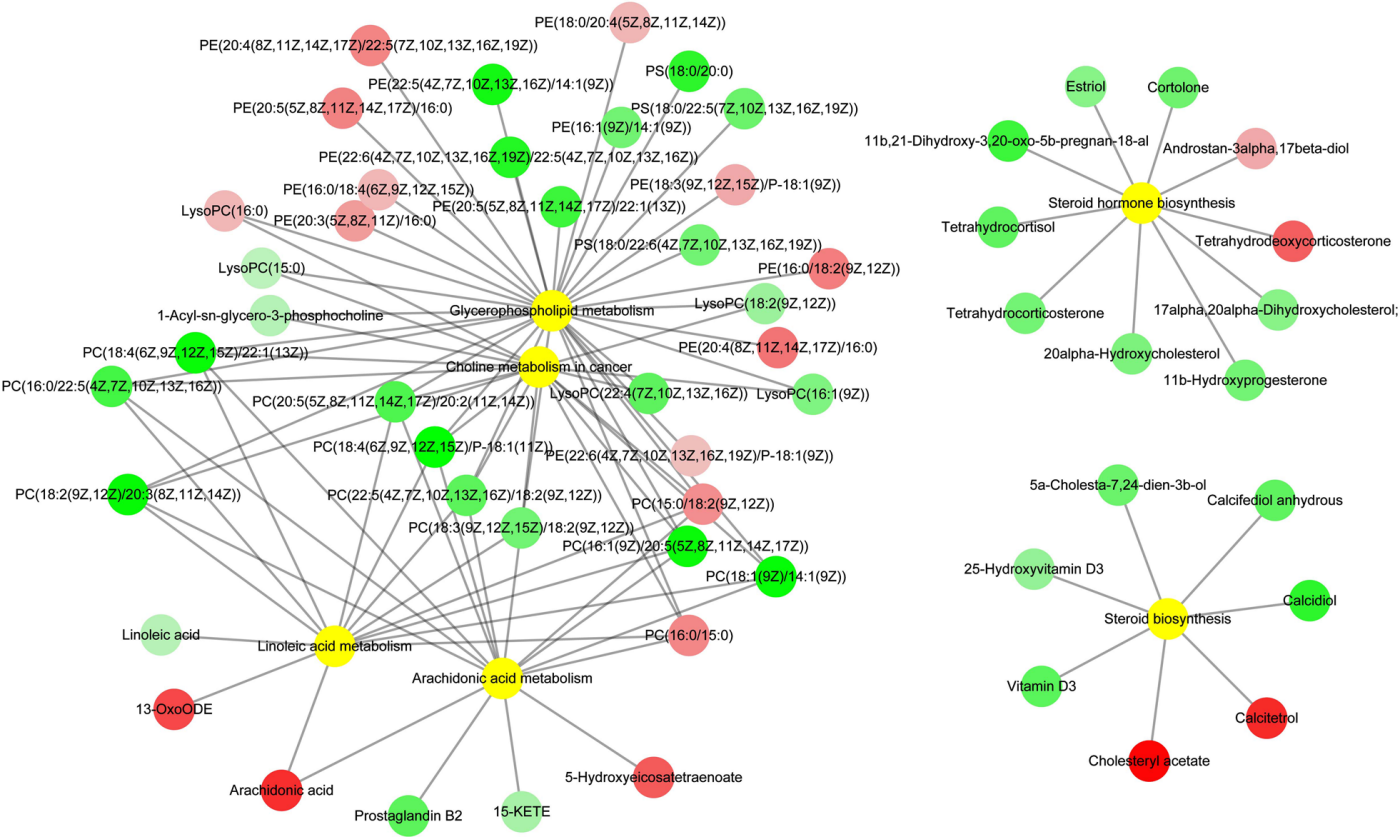
Metabolic pathways associated with downregulated metabolites at 21 dpi. Red, green and yellow colors represent upregulated metabolites, downregulated metabolites and the related metabolic pathways, respectively. *Abbreviations*: PS, phosphatidylserine; PC, phosphatidylcholine; PE, phosphatidylethanolamine; 13-OxoODE, (9Z,11E)-13-oxooctadeca-9,11-dienoic acid; 15-KETE, (5Z,8Z,11Z,13E)-15-oxoicosa-5,8,11,13-tetraenoic acid

As shown in Fig. 3b, the differentially abundant metabolites were involved in 25, 37 and 64 pathways, of which 6, 5 and 25 were unique at 7, 14 and 21 dpi, respectively. Twelve pathways were altered at all three sampling time points, namely metabolic pathways, biosynthesis of unsaturated fatty acids, glycerophospholipid metabolism, choline metabolism in cancer, regulation of autophagy, retrograde endocannabinoid signaling, primary bile acid biosynthesis, steroid hormone biosynthesis, arachidonic acid (AA) metabolism, linoleic acid metabolism, steroid biosynthesis and glycosylphosphatidylinositol (GPI)-anchor biosynthesis. Metabolites in the biosynthesis of unsaturated fatty acids pathway was upregulated as the infection progresses (Fig. 4). Metabolites in six pathways were downregulated at 21 dpi (Table 1 and Fig. 5).

**Table 1:** Metabolic pathways involving differentially abundant metabolites in mouse cerebral cortex at 7, 14 and 21 days after *T. gondii* infection

Pathway	7 dpi		14 dpi		21 dpi	
	Down	Up	Down	Up	Down	Up
Glycerophospholipid metabolism	0	6	3	2	21	12
Choline metabolism in cancer	0	3	0	2	14	3
Steroid hormone biosynthesis	0	1	0	2	8	2
Arachidonic acid metabolism	0	2	0	1	11	4
Linoleic acid metabolism	0	2	0	2	10	4
Steroid biosynthesis	0	1	1	1	5	2

### Identification of *T. gondii* responsive metabolites

**Fig. 6:**
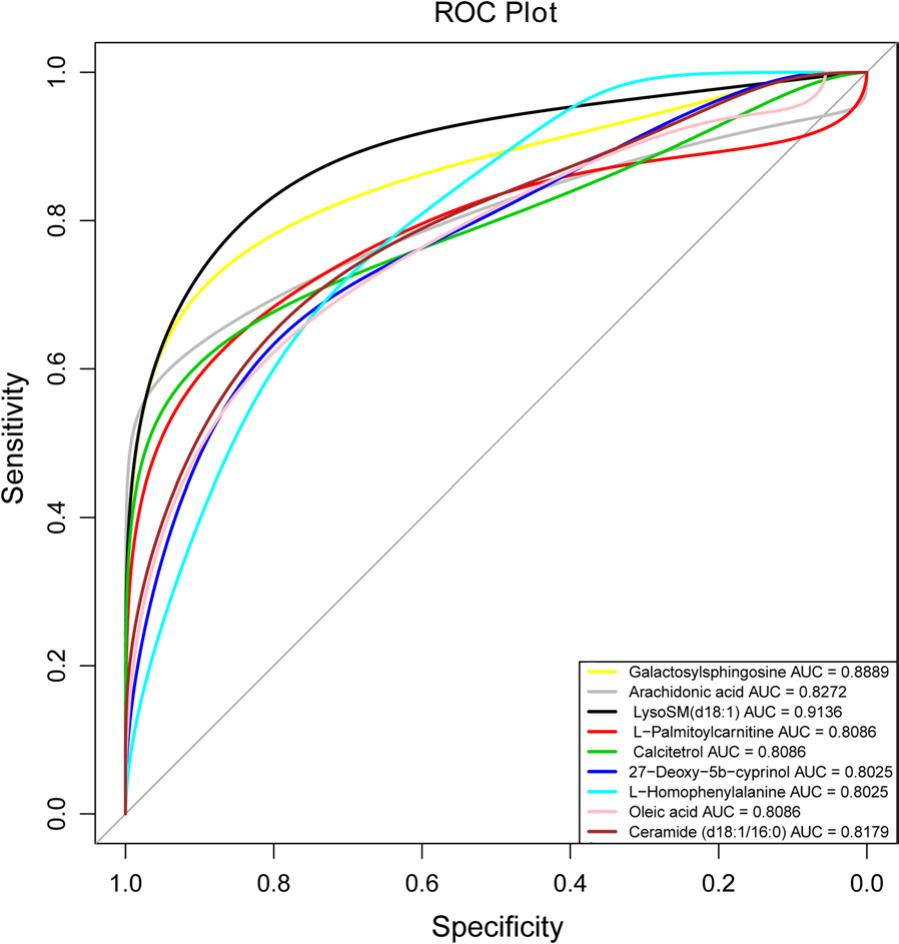
Receiver operating characteristic (ROC) curve showing *T. gondii* responsive metabolites. The x- and y-axes show false positive rate and true positive rate, respectively. *Abbreviation*: AUC, area under the curve

The results of ROC curve analysis revealed nine differentially abundant metabolites that had an AUC value > 0.8 and these were identified as *T. gondii* responsive metabolites: galactosylsphingosine, AA, LysoSM(d18:1), l-palmitoylcarnitine, calcitetrol, 27-Deoxy-5b-cyprinol, l-homophenylalanine, oleic acid and ceramide (d18:1/16:0) (Fig. 6). Among these, LysoSM (d18:1) was upregulated at 14 dpi; five metabolites (galactosylsphingosine, AA, l-palmitoylcarnitine, calcitetrol and 27-Deoxy-5b-cyprinol) exhibited differential abundance at 14 and 21 dpi, while the remaining three responsive metabolites [L-homophenylalanine, oleic acid and ceramide (d18:1/16:0)] showed differential abundance at 21 dpi. All these *T. gondii* responsive metabolites were upregulated (Additional file 2: Table S1).

## Discussion

Previous research studies have indicated that *T. gondii* can alter host behavior through manipulation of signaling pathways in the brain [22, 24]. However, the metabolic pathways involved in this process are only partially understood. Given that dysfunction of cerebral cortex underpins the neurological changes seen during *T. gondii* infection [25], the present study was set to characterize the metabolic changes of mouse cerebral cortex following *T. gondii* infection using a LC-MS-based metabolomics approach.

### Metabolic changes in cerebral cortex following *T. gondii* infection

The metabolic profiles of infected cerebral cortices were different from non-infected cerebral cortices. PLS-DA analysis revealed that metabolic differences between mouse groups became more evident as the infection progressed from 7 to 14 and 21 dpi. In the negative ionization mode, 64, 48 and 288 metabolites were differentially abundant at 7, 14 and 21 dpi, respectively. Additionally, 68, 54 and 229 differentially abundant metabolites were detected in the positive ionization mode. Although many differentially abundant metabolites were observed in the present study, only 22, 21 and 92 differentially abundant metabolites were mapped to metabolic pathways in the KEGG database at 7, 14 and 21 dpi, respectively (Fig. 3a). The differentially abundant metabolites were involved in 25, 37 and 64 pathways at 7, 14 and 21 dpi, respectively (Fig. 3b). Although only one common differentially expressed metabolite was found at all sampling times (Fig. 3a), 12 pathways were commonly altered in all collected cerebral cortices at all time points after infection (Fig. 3b).

### Time-dependent increase in the upregulated metabolites

Lipids play essential roles in the pathogenesis of *T. gondii* infection; parasite replication can be limited by fatty acid biosynthesis and availability [26]. Some fatty acid biosynthesis systems exist in the apicoplast and endoplasmic reticulum of *T. gondii* [27]. *Toxoplasma gondii* also requires acquisition of certain fatty acids from host cells. The host mitochondrial fusion can restrict *T. gondii* growth by limiting the uptake of fatty acids from the host cell [28]. In our study, the metabolites associated with biosynthesis of unsaturated fatty acid pathway were upregulated in the infected samples. Previous studies showed that N-3 polyunsaturated fatty acids and monounsaturated fatty acids reduce tissue responsiveness to cytokines, whereas n-6 polyunsaturated fatty acids [29] and saturated fatty acids [30] enhance the tissue inflammatory response. Given the inflammatory role of AA [31]; the immune-regulatory activity of linoleic acid [32]; the ability of stearic acid and palmitic acid to enhance the nuclear translocation of NF-κB p65 and induction of proinflammatory response [33]; the anti-inflammatory activity of docosahexaenoic acid in humans [34]; the synergistic effect of docosapentaenoic acid on the antiinflammatory function of docosahexaenoic acid [35]; and the inversely proportional relationship of oleic acid with natural killer cell activity [36], which is essential for *T. gondii* eradication [35], it is tempting to speculate that unsaturated fatty acids contribute to the regulation of host immune responses to *T. gondii* infection.

Noteworthy in our study is the upregulation of the immune response mediators (n-6 polyunsaturated fatty acids, such as AA and linoleic acid) and immune response antagonist (n-3 polyunsaturated fatty acids, such as docosahexaenoic acid and docosapentaenoic acid) at 14 dpi. We also found that at 21 dpi the immune response mediators, including one upregulated n-6 polyunsaturated fatty acids (AA), two upregulated saturated fatty acids (stearic acid and palmitic acid) and one downregulated n-6 polyunsaturated fatty acid (linoleic acid), were observed in the infected cerebral cortices. Additionally, two inflammatory antagonists, n-3 polyunsaturated fatty acid (docosahexaenoic acid-n3) and monounsaturated fatty acid (oleic acid), were upregulated. Furthermore, both oleic acid (inflammatory antagonist) and AA (immune response mediator) were identified as *T. gondii* responsive metabolites in the mouse cerebral cortex. These differential changes in the metabolites involved in the biosynthesis of unsaturated fatty acids in the cerebral cortex during *T. gondii* infection (Fig. 4) confirm previous studies [29, 30], suggesting immune-regulatory roles of unsaturated fatty acids during infection with this parasite. Interestingly, all these perturbed fatty acids are products of acyl-CoA thioesterase, indicating that acyl-CoA thioesterases play essential roles in mediating the interaction between *T. gondii* and mouse cerebral cortex.

### Downregulated metabolites in cerebral cortex

Out of the 12 pathways that were detected in our study, six included downregulated metabolites at 21 dpi, involved in glycerophospholipid metabolism, choline metabolism in cancer, steroid hormone biosynthesis, AA metabolism, linoleic acid metabolism and steroid biosynthesis. Some of these pathways participate in the regulation of neurological functions such as glycerophospholipid metabolism, steroid hormone biosynthesis and AA metabolism. The notion that *T. gondii* has a significant influence on the host behavior has been widely reported; however, the exact molecular mechanisms that link *T. gondii* infection to behavioral changes remain poorly defined. The membrane of neuronal cell is rich in glycerophospholipid, which is involved in the regulation of several molecular functions, such as generation of second messengers, apoptosis, antioxidant and membrane fusion, and regulation of enzyme activities [37]. Steroid hormone metabolites are barbiturate-like ligands of gamma-aminobutyric acid (GABA) receptor [38], which are expressed on the membrane of neurons and are downregulated by *T. gondii.* Alteration of AA metabolism has been implicated in neurological and psychiatric disorders [39]. In the present study, 21, 8, 5 and 11 metabolites involved in glycerophospholipid metabolism, steroid hormone biosynthesis, steroid biosynthesis and AA metabolism pathways, respectively, were downregulated at 21 dpi. The downregulation of metabolites in these neural activity-related pathways during latent infection may contribute to host behavioral changes.

It is worth noting that steroid hormone controls cytokine production of several immune cells [40]. Once metabolized by enzymes such as cyclooxygenases (COXs), lipoxygenases (LOs) and cytochrome P450 monooxygenases (CYPs), AA breaks down into several biologically active substances which activate host inflammatory response [41]. Although AA was identified as a *T. gondii* responsive metabolite and was upregulated in the present study, the metabolites of AA metabolism pathway were downregulated. This agrees with previous data showing that the enzymes of AA pathway are downregulated in mouse liver infected with *T. gondii* [42]. Furthermore, although steroid hormone biosynthesis pathway and AA metabolism pathway were upregulated in the murine liver and spleen during chronic *T. gondii*, the AA metabolism pathway was downregulated in the liver of acutely infected mice [18, 19].

In addition to AA, eight other *T. gondii* responsive metabolites were identified, namely galactosylsphingosine, LysoSM(d18:1), l-palmitoylcarnitine, calcitetrol, 27-Deoxy-5b-cyprinol, l-homophenylalanine, oleic acid and ceramide (Fig. 6). The accumulation of galactosylsphingosine (also known as psychosine) can result in oligodendroglial cell death and neural signaling dysfunction [43], which influences an animal’s behavior. Galactosylsphingosine was upregulated at 14 and 21 dpi (Additional file 2: Table S1). Whether the upregulation of galactosylsphingosine in mouse cerebral cortex contributes to *T. gondii* influence on mouse behavioral changes remains to be investigated. Calcitetrol (also known as 1,25-dihydroxyvitamin D3) participates in the regulation of T_reg_ cells [44]. Ceramide and oleic acid were upregulated at 21 dpi. Oleic acid (a monounsaturated fatty acid) serves as an inflammatory antagonist. In contrast, ceramide activates NLRP3 inflammasome [45], which is required for the control of *T. gondii* in macrophages [46]. Although functions of 27-Deoxy-5b-cyprinol, l-palmitoylcarnitine and LysoSM(d18:1) in the nervous system or immune system have not been elucidated, they could play as yet unknown roles in mediating the interaction between *T. gondii* and the host cerebral cortex.

## Conclusions

To our knowledge, this study provides the first comprehensive characterization of cerebral cortex metabolome following *T. gondii* infection in mice. We identified 73, 67 and 276 differentially abundant metabolites, which were involved in 25, 37 and 64 pathways at 7, 14 and 21 dpi, respectively. Differentially abundant metabolites related to biosynthesis of unsaturated fatty acid pathway increased as the infection advances. Twelve perturbed pathways related to neural activity, such as biosynthesis pathways of steroid hormone and arachidonic acid metabolism, were detected in all cerebral cortices at 7, 14 and 21 dpi. Nine metabolites were identified as responsive to *T. gondii* infection. Further analysis of these metabolites will enable the identification of new factors involved in the pathogenesis of cerebral toxoplasmosis.

## Additional files

**Additional file 1: Figure S1.** Agarose gel electrophoresis of PCR amplicons after amplification of *T. gondii* B1 gene DNA from cerebral cortices of *T. gondii*-infected and uninfected mice. Gels were stained with ethidium bromide and DNA was visualized under UV. *Abbreviations*: M, DL1000 DNA marker (TaKaRa, China); P, positive control; N, negative control.

**Additional file 2: Table S1.** Differentially expressed metabolites that were mapped to the KEGG pathways.

## Data Availability

The datasets supporting the findings of this article are included within the paper. The metabolomics data have been deposited in the MetaboLights database (http://www.ebi.ac.uk/metabolights) with accession number MTBLS770.

## Abbreviations

AA: arachidonic acid
GLT-1: glutamate transporter 1
LC–MS: liquid chromatography–mass spectrometry
SPF: specific-pathogen-free
PBS: phosphate-buffered saline
QC: quality control
SPE: solid phase extraction
UPLC: ultra-performance liquid chromatography
MS/MS: tandem mass spectrometry
HMDB: The Human Metabolome Database
KEGG: Kyoto Encyclopedia of Genes and Genomes
ESI−: negative electrospray ionization
ESI+: positive electrospray ionization
PLS-DA: partial least squares-discriminant analysis
ROC: receiver operating characteristic
AUC: area under the curve
GPI: glycosylphosphatidylinositol
