# Supplementary figures and images for: Metabolomic signature of mouse cerebral cortex following Toxoplasma gondii infection
Source: Parasit Vectors. 2019 Jul 29;12:373. doi: 10.1186/s13071-019-3623-4 (PMC6664753; doi:10.1186/s13071-019-3623-4)

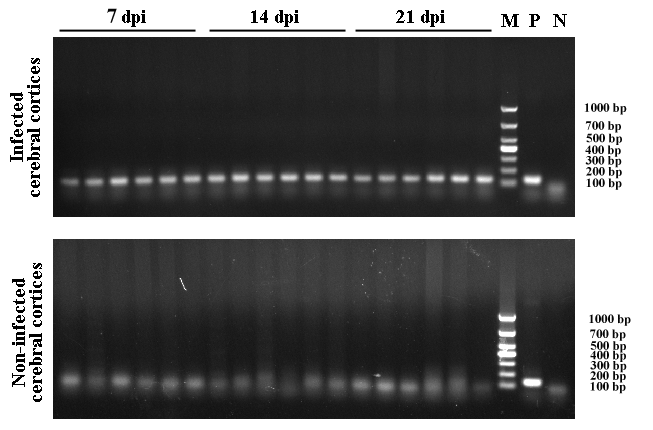

Supplement: Supplementary file 1 — Additional file 1: Figure S1. Agarose gel electrophoresis of PCR amplicons after amplification of T. gondii B1 gene DNA from cerebral cortices of T. gondii-infected and uninfected mice. Gels were stained with ethidium bromide and DNA was visualized under UV. Abbreviations: M, DL1000 DNA marker (TaKaRa, China); P, positive control; N, negative control. [file 13071_2019_3623_MOESM1_ESM.tif]
