# Supplementary material for: Metabolomic signature of mouse cerebral cortex following Toxoplasma gondii infection
Source: Parasit Vectors. 2019 Jul 29;12:373. doi: 10.1186/s13071-019-3623-4 (PMC6664753; doi:10.1186/s13071-019-3623-4)
Supplement: Supplementary file 2 — Additional file 2: Table S1. Differentially expressed metabolites that were mapped to the KEGG pathways. [file 13071_2019_3623_MOESM2_ESM.pdf]

Additional file 2: Table S1 Differentially abundant metabolites mapped to KEGG pathways.

Description *	Ratio	*P* value of *t*-test	VIP	m.z	Retention.time.min.	Pathway
**7 dpi NEG**						
4,5-Dihydroxyphthalate	1.627988	0.023925	1.469355	232.9855	0.519033	Metabolic pathways
13(1)-Hydroxy-magnesium-protoporphyrin IX 13-monomethyl ester	0.621275	0.047102	1.5604	649.2038	10.0284	Porphyrin and chlorophyll metabolism
Icosenoic acid	0.384702	0.040767	2.474858	309.2794	10.44915	Biosynthesis of unsaturated fatty acids
1-Acyl-sn-glycero-3-phosphocholine	1.623334	0.037207	1.433007	606.3297	9.693367	Glycerophospholipid metabolism; Choline metabolism in cancer
Catharanthine	0.809691	0.046837	1.034806	371.1535	9.850517	Metabolic pathways
2'-N-Acetylparomamine	0.695127	0.007028	1.493083	400.1503	9.956967	Butirosin and neomycin biosynthesis
Heme	0.778691	0.040871	1.202707	615.1703	9.97125	Porphyrin and chlorophyll metabolism
**7 dpi POS**						
2,4-Dichlorobenzoate	0.763986	0.029192	1.310524	172.9543	0.619033	Metabolic pathways
Glycolaldehyde	1.39997	0.038267	1.751688	98.98437	0.7112	Pentose and glucuronate interconversions; Glyoxylate and dicarboxylate metabolism; Vitamin B6 metabolism; Folate biosynthesis; Metabolic pathways
Methylimidazoleacetic acid	1.418866	0.028573	1.57542	123.0555	0.768333	Histidine metabolism
PE(14:0/22:6(4Z,7Z,10Z,13Z,16Z,19Z))	1.214774	0.031813	1.609063	774.4436	10.38147	Glycosylphosphatidylinositol(GPI)-anchor biosynthesis; Glycerophospholipid metabolism; Metabolic pathways; Regulation of

						autophagy; Retrograde endocannabinoid signaling
PE(22:1(13Z)/20:5(5Z,8Z,11Z,14Z,17Z))	1.844053	0.03692	2.146698	842.5679	10.52432	Glycosylphosphatidylinositol(GPI)-anchor biosynthesis; Glycerophospholipid metabolism; Metabolic pathways; Regulation of autophagy; Retrograde endocannabinoid signaling
7a-Hydroxy-cholestene-3-one	0.633277	0.036309	2.703883	418.3695	10.62433	Primary bile acid biosynthesis; Metabolic pathways
20alpha,22beta-Dihydroxycholesterol	1.297128	0.049305	1.445506	401.3408	10.64505	Steroid hormone biosynthesis; Metabolic pathways
PE(15:0/18:4(6Z,9Z,12Z,15Z))	1.240885	0.031949	1.481116	715.4981	10.85935	Glycosylphosphatidylinositol(GPI)-anchor biosynthesis; Glycerophospholipid metabolism; Metabolic pathways; Regulation of autophagy; Retrograde endocannabinoid signaling
PC(20:3(5Z,8Z,11Z)/14:0)	1.489598	0.022003	2.127636	778.5368	11.08008	Glycerophospholipid metabolism; Arachidonic acid metabolism; Linoleic acid metabolism; alpha-Linolenic acid metabolism; Metabolic pathways; Retrograde endocannabinoid signaling; Choline metabolism in cancer
PC(18:1(11Z)/16:0)	1.362562	0.036783	1.85845	760.5847	11.9316	Glycerophospholipid metabolism; Arachidonic acid metabolism;

						Linoleic acid metabolism; alpha-Linolenic acid metabolism; Metabolic pathways; Retrograde endocannabinoid signaling; Choline metabolism in cancer
Dodecanoic acid	1.893665	0.018439	2.594525	218.2118	5.4237	Fatty acid biosynthesis; Metabolic pathways
16-Hydroxypalmitate	1.842528	0.032996	2.798105	290.269	6.285933	Metabolic pathways
Calcidiol	1.436363	0.032356	1.541254	383.3292	9.210067	Steroid biosynthesis; Metabolic pathways Tuberculosis
beta-D-3-Ribofuranosyluric acid	0.769	0.025819	1.756768	301.0756	9.589117	Purine metabolism
Chitobiose	0.604085	0.035142	2.334956	449.1373	9.624767	Amino sugar and nucleotide sugar metabolism; Metabolic pathways; ABC transporters
**14 dpi NEG**						
PE(20:5(5Z,8Z,11Z,14Z,17Z)/16:0)	0.519879	0.006903	2.94358	736.4946	11.2335	Glycosylphosphatidylinositol(GPI)-anchor biosynthesis; Glycerophospholipid metabolism; Metabolic pathways; Regulation of autophagy; Retrograde endocannabinoid signaling
Galactosylsphingosine	2.744695	0.002289	3.684009	460.3277	7.768917	Sphingolipid metabolism
Arachidonate	2.394818	0.000211	3.839212	303.2325	8.003933	Arachidonic acid metabolism; Linoleic acid metabolism; Biosynthesis of unsaturated fatty acids; Metabolic pathways; Vascular smooth muscle contraction; Platelet

						activation; Fc epsilon RI signaling pathway; Fc gamma R-mediated phagocytosis; Retrograde endocannabinoid signaling; Serotonergic synapse; Long-term depression; Inflammatory mediator regulation of TRP channels; GnRH signaling pathway; Ovarian steroidogenesis; Oxytocin signaling pathway; Regulation of lipolysis in adipocytes; Aldosterone synthesis and secretion; Leishmaniasis; Amoebiasis
Docosahexaenoic acid	1.871345	0.038729	2.801723	327.23	8.003933	Biosynthesis of unsaturated fatty acids
1-Acyl-sn-glycero-3-phosphocholine	1.240363	0.020282	1.236428	602.3029	8.874	Glycerophospholipid metabolism; Choline metabolism in cancer
Linoleic acid	1.387929	0.034254	1.55048	279.2322	9.6505	Linoleic acid metabolism; Biosynthesis of unsaturated fatty acids; Metabolic pathways
17alpha,21-Dihydroxypregnenolone;	1.568237	0.021043	2.111805	347.2201	9.6505	Steroid hormone biosynthesis
Docosapentaenoic acid	1.401332	0.037032	1.398311	329.2478	9.7648	Biosynthesis of unsaturated fatty acids
Dolichyl b-D-glucosyl phosphate	1.313004	0.032799	1.293131	465.2241	9.850517	N-Glycan biosynthesis; Metabolic pathways

14 dpi POS

Salicin	0.759485	0.004257	2.154016	269.104	10.35263	Glycolysis / Gluconeogenesis; Taste transduction
PE(22:5(4Z,7Z,10Z,13Z,16Z)/16:0)	0.759897	0.039735	2.064293	766.5381	11.66915	Glycosylphosphatidylinositol(GPI)-anchor biosynthesis; Glycerophospholipid metabolism; Metabolic pathways; Regulation of autophagy; Retrograde endocannabinoid signaling
PE(18:4(6Z,9Z,12Z,15Z)/P-18:0)	0.725113	0.011434	2.261101	724.5269	11.77027	Glycosylphosphatidylinositol(GPI)-anchor biosynthesis; Glycerophospholipid metabolism; Metabolic pathways; Regulation of autophagy; Retrograde endocannabinoid signaling
LysoSM(d18:1)	2.756982	0.000252	4.973136	466.3525	7.854633	Sphingolipid metabolism
L-Palmitoylcarnitine	2.067998	0.000627	4.358549	400.3425	8.08965	Fatty acid degradation; Fatty acid metabolism
Calcitetrol	3.382591	2.40E-05	5.869029	450.3577	8.132517	Steroid biosynthesis; Metabolic pathways
27-Deoxy-5b-cyprinol	2.392092	0.010509	4.237968	454.389	8.524683	Primary bile acid biosynthesis; Metabolic pathways
LysoPC(18:2(9Z,12Z))	1.332001	0.005991	1.617473	520.3401	8.7597	Glycerophospholipid metabolism; Choline metabolism in cancer
2-Arachidonylglycerol	1.789272	0.021758	3.150968	401.2638	9.210067	Neuroactive ligand-receptor interaction; Retrograde endocannabinoid signaling
2-Methoxyestrone 3-glucuronide	1.488657	0.007734	2.417277	459.2046	9.273867	Steroid hormone biosynthesis

Vitamin A	1.247661	0.044233	1.719875	287.2351	9.28815	Retinol metabolism; Metabolic pathways; Vitamin digestion and absorption
5a-Cholesta-7,24-dien-3b-ol	0.751589	0.010887	1.151147	385.3466	9.6536	Steroid biosynthesis; Metabolic pathways
**21 dpi NEG**						
Beta-D-Fructose 6-phosphate	2.982175	0.001827	2.615302	259.0222	0.633333	Glycolysis / Gluconeogenesis; Pentose phosphate pathway; Fructose and mannose metabolism; Amino sugar and nucleotide sugar metabolism; Metabolic pathways; Carbon metabolism; Biosynthesis of amino acids
Xanthine	1.564588	0.029563	1.518773	151.0249	0.76835	Purine metabolism; Caffeine metabolism; Metabolic pathways
3alpha,7alpha,12alpha-Trihydroxy-5beta-cholestan-26-al	1.615534	0.041478	1.859227	433.3321	10.18555	Primary bile acid biosynthesis; Metabolic pathways
PC(18:4(6Z,9Z,12Z,15Z)/P-18:1(11Z))	0.141297	0.001609	4.213948	798.5254	10.30628	Glycerophospholipid metabolism; Arachidonic acid metabolism; Linoleic acid metabolism; alpha-Linolenic acid metabolism; Metabolic pathways; Retrograde endocannabinoid signaling; Choline metabolism in cancer
Dermatan	1.205861	0.022492	1.097477	551.1052	10.34913	Glycosaminoglycan degradation; Metabolic pathways; Proteoglycans in cancer

PE(22:6(4Z,7Z,10Z,13Z,16Z,19Z)/22:5(4Z,7Z,10Z,13Z,16Z))	0.356982	0.013213	2.883902	836.5249	10.36343	Glycosylphosphatidylinositol(GPI)-anchor biosynthesis; Glycerophospholipid metabolism; Metabolic pathways; Regulation of autophagy; Retrograde endocannabinoid signaling
PC(18:2(9Z,12Z)/20:3(8Z,11Z,14Z))	0.205772	0.008205	3.213622	842.5494	10.52058	Glycerophospholipid metabolism; Arachidonic acid metabolism; Linoleic acid metabolism; alpha-Linolenic acid metabolism; Metabolic pathways; Retrograde endocannabinoid signaling; Choline metabolism in cancer
PC(18:1(9Z)/14:1(9Z))	0.192117	0.001763	3.433333	764.5011	10.71273	Glycerophospholipid metabolism; Arachidonic acid metabolism; Linoleic acid metabolism; alpha-Linolenic acid metabolism; Metabolic pathways; Retrograde endocannabinoid signaling; Choline metabolism in cancer
PC(18:4(6Z,9Z,12Z,15Z)/22:1(13Z))	0.216445	0.002483	3.452981	870.5855	10.88418	Glycerophospholipid metabolism; Arachidonic acid metabolism; Linoleic acid metabolism; alpha-Linolenic acid metabolism; Metabolic pathways; Retrograde endocannabinoid signaling; Choline metabolism in cancer

PE(20:5(5Z,8Z,11Z,14Z,17Z)/16:0)	1.560694	0.038885	1.439676	736.4946	11.2335	Glycosylphosphatidylinositol(GPI)-anchor biosynthesis; Glycerophospholipid metabolism; Metabolic pathways; Regulation of autophagy; Retrograde endocannabinoid signaling
PE(20:4(8Z,11Z,14Z,17Z)/22:5(7Z,10Z,13Z,16Z,19Z))	1.550398	0.021245	1.745249	812.5266	11.43995	Glycosylphosphatidylinositol(GPI)-anchor biosynthesis; Glycerophospholipid metabolism; Metabolic pathways; Regulation of autophagy; Retrograde endocannabinoid signaling
PE(20:4(8Z,11Z,14Z,17Z)/16:0)	1.654044	0.018951	1.723681	738.5095	11.55423	Glycosylphosphatidylinositol(GPI)-anchor biosynthesis; Glycerophospholipid metabolism; Metabolic pathways; Regulation of autophagy; Retrograde endocannabinoid signaling
PE(16:0/18:2(9Z,12Z))	1.588417	0.00634	1.747393	714.51	11.5828	Glycosylphosphatidylinositol(GPI)-anchor biosynthesis; Glycerophospholipid metabolism; Metabolic pathways; Regulation of autophagy; Retrograde endocannabinoid signaling
PE(20:3(5Z,8Z,11Z)/16:0)	1.40368	0.030547	1.416828	740.525	11.68925	Glycosylphosphatidylinositol(GPI)-anchor biosynthesis; Glycerophospholipid metabolism;

						Metabolic pathways; Regulation of autophagy; Retrograde endocannabinoid signaling
PE(16:0/18:4(6Z,9Z,12Z,15Z))	1.256173	0.012089	1.042306	746.4489	11.71782	Glycosylphosphatidylinositol(GPI)-anchor biosynthesis; Glycerophospholipid metabolism; Metabolic pathways; Regulation of autophagy; Retrograde endocannabinoid signaling
PC(16:0/15:0)	1.531811	0.006452	1.0216	718.54	11.78925	Glycerophospholipid metabolism; Arachidonic acid metabolism; Linoleic acid metabolism; alpha-Linolenic acid metabolism; Metabolic pathways; Retrograde endocannabinoid signaling; Choline metabolism in cancer
PC(15:0/18:2(9Z,12Z))	1.487998	0.013967	1.395747	742.5411	12.01012	Glycerophospholipid metabolism; Arachidonic acid metabolism; Linoleic acid metabolism; alpha-Linolenic acid metabolism; Metabolic pathways; Retrograde endocannabinoid signaling; Choline metabolism in cancer
L-Homophenylalanine	1.201077	0.006145	1.173437	178.0865	3.384967	Phenylalanine, tyrosine and tryptophan biosynthesis; 2-Oxocarboxylic acid metabolism
Galactosylsphingosine	4.772794	1.72E-05	4.211398	460.3277	7.768917	Sphingolipid metabolism

Arachidonate	2.572414	0.000503	3.220142	303.2325	8.003933	Arachidonic acid metabolism; Linoleic acid metabolism; Biosynthesis of unsaturated fatty acids; Metabolic pathways; Vascular smooth muscle contraction; Platelet activation; Fc epsilon RI signaling pathway; Fc gamma R-mediated phagocytosis; Retrograde endocannabinoid signaling; Serotonergic synapse; Long-term depression; Inflammatory mediator regulation of TRP channels; GnRH signaling pathway; Ovarian steroidogenesis; Oxytocin signaling pathway; Regulation of lipolysis in adipocytes; Aldosterone synthesis and secretion; Leishmaniasis; Amoebiasis
Docosahexaenoic acid	2.034204	0.005471	2.411878	327.23	8.003933	Biosynthesis of unsaturated fatty acids
Palmitic acid	2.559633	0.014517	2.998377	255.2324	8.10395	Fatty acid biosynthesis; Fatty acid elongation; Fatty acid degradation; Biosynthesis of unsaturated fatty acids; Metabolic pathways; Fatty acid metabolism
Oleic acid	3.618674	0.004044	3.681572	281.2483	8.218233	Fatty acid biosynthesis; Biosynthesis of unsaturated fatty acids

LysoPC(16:1(9Z))	0.637513	0.003387	1.780251	492.3102	8.951883	Glycerophospholipid metabolism; Choline metabolism in cancer
LysoPC(16:0)	1.256671	0.011725	1.146358	530.3052	9.066167	Glycerophospholipid metabolism; Choline metabolism in cancer
5-Hydroxyeicosatetraenoate	1.888633	0.017928	1.997717	319.2275	9.151883	Arachidonic acid metabolism; Inflammatory mediator regulation of TRP channels
Divinylchlorophyll a	0.751285	0.020723	1.201267	889.5156	9.515483	Porphyrin and chlorophyll metabolism
Stearic acid	1.937012	0.012259	2.401965	283.2631	9.55835	Fatty acid biosynthesis; Biosynthesis of unsaturated fatty acids
1-Acyl-sn-glycero-3-phosphocholine	0.543907	0.006163	1.876112	606.3297	9.693367	Glycerophospholipid metabolism; Choline metabolism in cancer
PE(16:1(9Z)/14:1(9Z))	0.59636	0.003791	1.858339	694.4255	9.836233	Glycosylphosphatidylinositol(GPI)-anchor biosynthesis; Glycerophospholipid metabolism; Metabolic pathways; Regulation of autophagy; Retrograde endocannabinoid signaling
PE(22:5(4Z,7Z,10Z,13Z,16Z)/14:1(9Z))	0.293538	0.000295	3.629551	770.4542	9.836233	Glycosylphosphatidylinositol(GPI)-anchor biosynthesis; Glycerophospholipid metabolism; Metabolic pathways; Regulation of autophagy; Retrograde endocannabinoid signaling
PC(16:1(9Z)/20:5(5Z,8Z,11Z,14Z,17Z))	0.244134	0.000167	3.668308	812.5064	9.956967	Glycerophospholipid metabolism; Arachidonic acid metabolism;

						Linoleic acid metabolism; alpha-Linolenic acid metabolism; Metabolic pathways; Retrograde endocannabinoid signaling; Choline metabolism in cancer
3-O-Sulfogalactosylceramide (d18:1/16:0)	0.441758	0.03554	2.472167	778.5211	9.985533	Sphingolipid metabolism; Metabolic pathways
**21 dpi POS**						
5a-Cholesta-7,24-dien-3b-ol	0.572883	0.001706	3.028826	385.3447	10.2319	Steroid biosynthesis; Metabolic pathways
Ubiquinone-8	0.69587	0.002134	2.809335	765.5171	10.35263	Ubiquinone and other terpenoid-quinone biosynthesis
PC(18:3(9Z,12Z,15Z)/18:2(9Z,12Z))	0.59141	0.004071	2.974271	802.5319	10.35263	Glycerophospholipid metabolism; Arachidonic acid metabolism; Linoleic acid metabolism; alpha-Linolenic acid metabolism; Metabolic pathways; Retrograde endocannabinoid signaling; Choline metabolism in cancer
Calcidiol	0.39057	0.001459	3.782512	401.3412	10.46718	Steroid biosynthesis; Metabolic pathways; Tuberculosis
PE(20:5(5Z,8Z,11Z,14Z,17Z)/22:1(13Z))	0.439191	0.001948	3.558609	842.5679	10.52432	Glycosylphosphatidylinositol(GPI)-anchor biosynthesis; Glycerophospholipid metabolism; Metabolic pathways; Regulation of autophagy; Retrograde endocannabinoid signaling

PS(18:0/22:5(7Z,10Z,13Z,16Z,19Z))	0.573796	0.005104	2.836821	820.5478	10.59575	Glycine, serine and threonine metabolism; Glycerophospholipid metabolism; Metabolic pathways; Leishmaniasis; Amoebiasis; Systemic lupus erythematosus
25-Hydroxyvitamin D3	0.693377	0.003268	2.411821	423.3258	10.6279	Steroid biosynthesis; Metabolic pathways; Tuberculosis
17alpha,20alpha-Dihydroxycholesterol;	0.664756	0.001248	2.643533	401.3408	10.64505	teroid hormone biosynthesis
PS(18:0/20:0)	0.385932	0.002002	3.752662	842.5805	10.67363	Glycine, serine and threonine metabolism; Glycerophospholipid metabolism; Metabolic pathways; Leishmaniasis; Amoebiasis; Systemic lupus erythematosus
Vitamin D3	0.529468	0.000949	3.110825	385.3463	10.8165	Steroid biosynthesis; Metabolic pathways; Vitamin digestion and absorption; Rheumatoid arthritis
20alpha-Hydroxycholesterol	0.62723	0.002886	2.561981	425.339	10.8165	Steroid hormone biosynthesis; Metabolic pathways
SM(d18:0/16:1(9Z))	2.2955	0.004374	3.878188	703.5744	11.29447	Sphingolipid metabolism; Metabolic pathways; Sphingolipid signaling pathway
Ceramide (d18:1/16:0)	1.832374	0.002591	2.635071	520.5092	11.60538	Sphingolipid metabolism; Metabolic pathways; Sphingolipid signaling pathway; Neurotrophin signaling pathway; Adipocytokine signaling pathway; Insulin resistance; AGE-

						RAGE signaling pathway in diabetic complications; Leishmaniasis
Cholesteryl acetate	4.762766	0.002405	5.537349	429.3732	11.71268	Steroid biosynthesis; Ovarian steroidogenesis; Fat digestion and absorption; Bile secretion; Vitamin digestion and absorption
SM(d18:0/18:1(11Z))	2.068757	0.005533	3.580058	731.6066	11.75587	Sphingolipid metabolism; Metabolic pathways; Sphingolipid signaling pathway
PE(18:3(9Z,12Z,15Z)/P-18:1(9Z))	1.324946	0.00901	1.780657	724.5269	11.77027	Glycosylphosphatidylinositol(GPI)-anchor biosynthesis; Glycerophospholipid metabolism; Metabolic pathways; Regulation of autophagy; Retrograde endocannabinoid signaling
PE(22:6(4Z,7Z,10Z,13Z,16Z,19Z)/P-18:1(9Z))	1.221288	0.04759	1.288592	774.5393	11.82825	Glycosylphosphatidylinositol(GPI)-anchor biosynthesis; Glycerophospholipid metabolism; Metabolic pathways; Regulation of autophagy; Retrograde endocannabinoid signaling
PE(18:0/20:4(5Z,8Z,11Z,14Z))	1.267038	0.039195	1.889707	750.5431	11.87368	Glycosylphosphatidylinositol(GPI)-anchor biosynthesis; Glycerophospholipid metabolism; Metabolic pathways; Regulation of autophagy; Retrograde endocannabinoid signaling

Toluene	0.813193	0.023413	1.824521	93.07007	5.879467	Metabolic pathways
2'-Deamino-2'-hydroxyparomamine;	0.64466	0.016577	2.02378	307.1517	6.606667	Butirosin and neomycin biosynthesis
Pravastatin	0.733906	0.049423	1.620861	407.2386	7.113133	Bile secretion
Traumatin	0.713673	0.001754	2.676718	195.1372	7.19885	alpha-Linolenic acid metabolism
S-(2-Hydroxyethyl)-N-acetyl-L-cysteine;	0.800495	0.00539	1.666194	208.0629	7.19885	Metabolism of xenobiotics by cytochrome P450
2-Hydroxy-6-oxo-6-(2-hydroxyphenoxy)-hexa-2,4-dienoate;	0.632226	0.000212	2.947256	251.0561	7.202433	Metabolic pathways
Xanthoxin	0.816202	0.005225	1.730667	251.1624	7.597467	Metabolic pathways
Neomycin	0.650174	0.043146	2.316403	597.3113	7.61175	Butirosin and neomycin biosynthesis
2-(3'-Methylthio)propylmalic acid	1.407361	0.001055	2.577576	261.0211	7.72605	2-Oxocarboxylic acid metabolism
FMNH2	1.322392	0.030885	1.73693	441.1209	7.72605	Riboflavin metabolism; Metabolic pathways
Cortolone	0.581532	0.025143	2.313912	389.2305	7.7832	Steroid hormone biosynthesis
Sphingosyl-phosphocholine	3.251218	0.000257	5.168744	466.3525	7.854633	Sphingolipid metabolism
Prostaglandin B2	0.533646	0.027738	2.334988	357.2037	7.886767	Arachidonic acid metabolism; Metabolic pathways; Serotonergic synapse
11b,21-Dihydroxy-3,20-oxo-5b-pregnan-18-al	0.439009	0.03179	2.636187	387.2153	7.9325	Steroid hormone biosynthesis
11b-Hydroxyprogesterone	0.607113	0.019871	2.153665	355.1875	7.946783	Steroid hormone biosynthesis
Estriol	0.655182	0.00283	2.861777	289.1803	7.961067	Steroid hormone biosynthesis; Metabolic pathways
(9Z,11E,15Z)-(13S)-Hydroperoxyoctadeca-9,11,15-trienoate	0.484162	0.019553	2.674823	333.2042	7.961067	alpha-Linolenic acid metabolism; Metabolic pathways
Tetrahydrocortisol	0.546558	0.046217	2.296288	389.2302	8.003933	Steroid hormone biosynthesis
L-Palmitoylcarnitine	2.440759	9.37E-05	4.800594	400.3425	8.08965	Fatty acid degradation; Fatty acid metabolism

Calcitetrol	2.611776	0.012727	3.84392	450.3577	8.132517	Steroid biosynthesis; Metabolic pathways
2-Arachidonoylglycerol	0.447526	0.030793	2.841682	396.3098	8.381817	Neuroactive ligand-receptor interaction; Retrograde endocannabinoid signaling
9(S)-HOT; (9S)-(10E,12Z,15Z)-9-Hydroxyoctadecatri-10,12,15-enoic acid; 9(S)-HOTrE;	1.405098	0.005728	2.722594	277.2153	8.481817	alpha-Linolenic acid metabolism
13-OxoODE	2.104412	0.000461	3.73962	317.2083	8.481817	Linoleic acid metabolism
15-KETE	0.746043	0.006826	1.741688	301.2158	8.524683	Arachidonic acid metabolism
27-Deoxy-5b-cyprinol	3.992	0.000703	6.097428	454.389	8.524683	Primary bile acid biosynthesis; Metabolic pathways
Tetrahydrocorticosterone	0.600269	0.037094	2.141009	373.2346	8.538967	Steroid hormone biosynthesis
LysoPC(15:0)	0.813384	0.008698	1.502084	482.3244	8.802567	Glycerophospholipid metabolism; Choline metabolism in cancer
LysoPC(18:2(9Z,12Z))	0.714505	0.011555	2.016584	520.3399	8.888283	Glycerophospholipid metabolism; Choline metabolism in cancer
LysoPC(22:4(7Z,10Z,13Z,16Z))	0.788134	0.027996	1.402678	568.3403	8.888283	Glycerophospholipid metabolism; Choline metabolism in cancer
Androstan-3alpha,17beta-diol	1.329948	0.008666	2.095944	293.2467	8.923617	Steroid hormone biosynthesis
Linoleic acid	0.797409	0.011512	1.69308	298.2742	8.923617	Linoleic acid metabolism; Biosynthesis of unsaturated fatty acids; Metabolic pathways
Tetrahydrodeoxycorticosterone	1.835858	0.014289	3.257091	357.2399	9.02365	Steroid hormone biosynthesis
Tryptophol	0.820555	0.043699	1.373631	184.0737	9.195717	Tryptophan metabolism
Calcifediol anhydrous	0.549357	0.008247	2.957244	383.3292	9.210067	Steroid biosynthesis; Metabolic pathways; Tuberculosis

PC(22:5(4Z,7Z,10Z,13Z,16Z)/18:2(9Z,12Z))	0.533907	0.009299	3.107997	870.5337	9.6101	Glycerophospholipid metabolism; Arachidonic acid metabolism; Linoleic acid metabolism; alpha-Linolenic acid metabolism; Metabolic pathways; Retrograde endocannabinoid signaling; Choline metabolism in cancer
7alpha-Hydroxy-3-oxo-4-cholestenoate;	0.523604	0.00035	3.29407	431.3153	9.839317	Primary bile acid biosynthesis
PC(20:5(5Z,8Z,11Z,14Z,17Z)/20:2(11Z,14Z))	0.494452	0.002961	3.263398	870.5388	9.839317	Glycerophospholipid metabolism; Arachidonic acid metabolism; Linoleic acid metabolism; alpha-Linolenic acid metabolism; Metabolic pathways; Retrograde endocannabinoid signaling; Choline metabolism in cancer
Bacteriochlorophyll a	0.640671	0.020365	2.748601	911.5536	9.839317	Porphyrin and chlorophyll metabolism
3-O-Sulfogalactosylceramide (d18:1/18:0)	0.513492	0.002055	3.356449	846.5207	9.853683	Sphingolipid metabolism; Metabolic pathways
PC(16:0/22:5(4Z,7Z,10Z,13Z,16Z))	0.458292	0.000616	3.569629	846.5407	9.960233	Glycerophospholipid metabolism; Arachidonic acid metabolism; Linoleic acid metabolism; alpha-Linolenic acid metabolism; Metabolic pathways; Retrograde endocannabinoid signaling; Choline metabolism in cancer

PS(18:0/22:6(4Z,7Z,10Z,13Z,16Z,19Z))	0.597889	0.026969	2.680167	858.5318	9.839317	Glycine, serine and threonine metabolism; Glycerophospholipid metabolism; Metabolic pathways; Leishmaniasis; Amoebiasis; Systemic lupus erythematosus
Fexofenadine	0.573118	0.027832	2.890652	524.279	7.918217	Bile secretion

*POS and NEG indicate positive and negative ion mode, respectively; PS: phosphatidylserine; PC: phosphatidylcholine; PE: phosphatidylethanolamine; 13-OxoODE: (9Z,11E)-13-oxooctadeca-9,11-dienoic acid; 15-KETE: (5Z,8Z,11Z,13E)-15-oxoicosa-5,8,11,13-tetraenoic acid; SM, sphingomyelin; FMNH2: {[(2R,3S,4S)-5-{7,8-dimethyl-2,4-dioxo-1H,2H,3H,4H,5H,10H-benzo[g]pteridin-10-yl}-2,3,4-trihydroxypentyl]oxy}phosphonic acid.
